# Impact of a theory-informed and user-centered stroke information campaign on the public’s behaviors, attitudes, and knowledge when facing acute stroke: a controlled before-and-after study

**DOI:** 10.1186/s12889-020-09795-y

**Published:** 2020-11-16

**Authors:** Julie Haesebaert, Caroline Laude, Anne Termoz, Estelle Bravant, Nathalie Perreton, Thomas Bony, Hélène Trehard, Sylvie Porthault, Laurent Derex, Norbert Nighoghossian, Anne-Marie Schott

**Affiliations:** 1 Université de Lyon, Université Claude Bernard Lyon 1 - HESPER EA 7425, Lyon, France; 2 Hospices Civils de Lyon, Pôle Santé Publique, F-69003 Lyon, France; 3 Hospices Civils de Lyon, Hôpital Edouard Herriot, PAM Urgences Réanimation Médicales, Lyon, France; 4 Hospices Civils de Lyon, Hôpital Pierre Wertheimer, Stroke Center, Lyon, France

**Keywords:** Stroke, Awareness campaign, Patient attitude, Public education

## Abstract

**Background:**

Public awareness of stroke symptoms is a key factor to ensure access to reperfusion strategies in due time. We designed and launched a regional theory-informed and user-centered information campaign and assessed its impact on emergency medical services (EMS) calls for stroke suspicion, time-to-call, and public attitudes and awareness concerning stroke.

**Methods:**

A controlled before-and-after study was conducted during 3 sequential time-periods in 2 separate counties. Key messages of the campaign were underpinned by stroke representations and the theory of planned behavior, and focused on recognition of stroke warning signs and the need to call EMS urgently. The campaign included posters, leaflets, adverts and films displayed in bus and subway stations, internet, social networks, and local radio. Outcome measures on behavior, attitudes, and knowledge were assessed before the launch of the campaign, at 3 months, and 12 months.

**Results:**

The number of EMS calls for stroke suspicion increased by 21% at 12 months in the intervention county and this change was significantly different to that observed in the control county (*p* = 0.02). No significant changes were observed regarding self-reported attitudes in case of stroke. An 8% significant increase in recognizing at least 2 stroke warning signs was observed in the intervention county (*p* = 0.04) at 3 months, while it did not change significantly in the control county (*p* = 0.6). However, there was no significant difference in warning sign recognition between both counties (*p* = 0.16).

**Conclusion:**

The campaign significantly improved public’s behavior of calling EMS, although stroke knowledge was not improved as much as expected. Repeating these campaigns over time might further help improve timeliness and access to reperfusion strategies.

**Trial registration:**

Clinical Trial Registration-URL: http://www.clinicaltrials.gov. Unique identifier: NCT02846363.

**Supplementary Information:**

The online version contains supplementary material available at 10.1186/s12889-020-09795-y.

## Background

Knowledge concerning stroke symptoms is poor in the general population [1–3]. Patients and relatives thus encounter difficulties in symptom recognition, and response is often inappropriate. While it has been demonstrated that calling emergency medical services (EMS) immediately constitutes the best choice in case of stroke, the majority of patients delay their contact with medical services by going directly to emergency departments (ED) or general practitioners [4, 5]. These increased delays are incompatible with access to an effective stroke treatment and increase stroke morbidity and mortality [6]. In a previous cohort study in the Rhône county (France), less than 1 patient in 2 called EMS in case of stroke symptoms, 80% were managed in ED with a median time between symptom onset and admission over 2 h, and 8% were thrombolyzed [7]. With the aim of increasing access to thrombolysis, a number of actions targeting professionals and care organization have been carried out [8, 9]. Targeting the general population however, would also help improve stroke management by decreasing time from symptom onset to first medical contact and increase EMS calls.

Information campaigns may positively impact stroke knowledge, but they often lack theoretical framework, and their impact on behaviors remains low [10–12]. To develop a more effective campaign that fits the needs of the targeted population, we first carried out a study to assess the population’s representations of stroke [13]. Based on the results of this study and informed by the theory of planned behavior (TPB) [14], we developed the ReACT campaign which aimed to improve the skills of the general population in recognizing the first signs of acute stroke and to understand the need to promptly call EMS. The main objective of the present study was to assess the impact of the campaign on the number of calls to EMS for suspected stroke. The secondary objectives were to assess the effect of the campaign on time from symptom onset to EMS call, and the public’s knowledge of stroke in the intervention county compared to a control county.

## Methods

### Study design

We conducted a quasi-experimental controlled before-and-after study, comparing the Rhône county where the campaign was implemented (intervention county) to the Isère county (control county), a nearby region with no local specific campaign during the study period. The ReACT campaign was launched in the Rhône county from October 29th 2014 (World Stroke Day), for a 2-month period. The evaluation process occurred during three 2-month evaluation periods: before the launch of the campaign on World Stroke Day (T0); at 3 months (T1; short term); and at 12 months (T2; long term). Reporting of the results follows the TREND Statement [15]. No ethics approval was deemed necessary according to French regulation to conduct the study which was classified as non-interventional (3° of Article L.1121–1 of the French Public Health Code).

### Content of the campaign

The ReACT campaign was an information campaign which aimed to improve stroke recognition in the general population and prompt call to EMS. The content and the form of the messages were designed by a multidisciplinary team involving stroke unit (SU) neurologists, EMS physicians, public health researchers, pharmacists, psychologists, communication professionals, and representatives of patients’ associations, using a user-centered approach. The content of the campaign targeted three dimensions: stroke symptoms, urgency of the situation and need to call EMS. Information on stroke symptom recognition targeted the 3 FAST-symptoms (Face dropping, Arm weakness, Speech disturbance). The urgency of the situation and the need to call EMS were indicated in all campaign media. The content of the campaign was informed by the TPB [14] and based on public stroke representations [13] highlighted in a previous qualitative study conducted by our team which results showed a strong feeling of fatalism and a low perceived self-efficacy to act appropriately. It also pointed out the importance of lay knowledge and shared experiences to gain understanding concerning stroke. Based on these results, we designed the campaign presenting stroke symptoms in everyday-life using characters and situations with which everyone could identify. In line with TPB, the content of the campaign targeted social norms and perceived behavioral control, using a vicarious model. To target subjective norms, we presented stroke victims as well as non-professional rescuers, as both men and women, with various ages (from grandchildren to grandfather). To improve perceived behavioral control and self-efficacy, our campaign slogan was ‘Your call can save life’ and messages displayed mentioned that everyone can effectively act by just a phone call. All media were developed using 3 visuals: one young student female presenting sudden arm weakness, one middle aged working male presenting sudden speech disturbance, and one grand-father presenting sudden face weakness. In each, one relative effectively acted by immediately calling EMS which enabled the patient to be saved. The main diffusion media were posters, leaflets, and videos on a dedicated internet website. The leaflet included information on stroke risk factors, symptoms, acute management, and treatments at acute and chronic phase. We assessed its understandability, content and form in a random sample of patients identified through several general practitioners. The messages in the posters focused on self-efficacy reinforcement (“he/she saves his/her life”, “he/she just call the 15” -15 being the French equivalent for 911-“Your phone call can save lives”). In the movies, the same situation as presented in the posters were played, and the listeners could choose the end of the film by choosing whether they wanted to wait and see or immediately call EMS, such as they could understand benefit in rapid stroke management at acute phase. All campaign materials are provided in Appendix 1.

### Conduct of the campaign

A communication plan was designed specifically for our stroke campaign by communication professionals from a communication school. The campaign was led by the steering committee in partnership with the communication school. Several service providers were responsible for the distribution of the materials (radio campaign, poster campaign, website design). The diffusion strategy consisted in one public event on World Stroke Day in the city-center of Lyon, the biggest city of the county, followed by a 2-month multi-media campaign. The event proposed a press conference and an information booth animated by representatives of different health professionals involved in stroke management as well as stroke patients. A free risk-factor screening by a prevention nurse was also proposed in a bus, which included a brief individual interview, information, measure of arterial blood pressure, and capillary blood testing for glucose and cholesterol. On the street, actors performed short interactive theatrical scenes to engage people in discussing the response to adopt in case of stroke. The multimedia campaign following the event was composed of a poster campaign on 233 subway and bus stations of the Rhône county, a one-week advert broadcasting on the two main local radios, an information website, and a social network group on Facebook. The posters and leaflets of the campaign were sent to all the 3600 ambulatory physicians (general practitioners and all medical specialties) of the county and to 60 pharmacists. The posters were also displayed in the four university hospitals of the Rhône county.

### Patient involvement

Two patients’ representatives, from the local stroke patient association, were involved in the research, since the first step of the study, just after grant approval. They were involved in the design of the intervention, in the choice of the content of the information leaflet and campaign, to include their experience and preferences; they also participated in organizing the event on world stroke day. They critically revised the study questionnaires and participated in the spreading of the questionnaires for recruitment of participants.

### Population and geographic counties

The target population for the intervention was the general population of the Rhône county (1.8 million inhabitants) covered by two SU and one EMS organization (SAMU69). The control group was the nearby Isère county (1.2 million inhabitants) covered by two SU and one EMS organization (SAMU38). The Rhône/Isère populations had similar socio-demographic characteristics including 51.8%/50.8% of women, 58.2%/56.6% of population aged ≥65 years old, and 12.5%/11.2% unemployment rates [16]. Citizens living in these two counties and aged above 18 years old were eligible for the survey on stroke knowledge and attitudes. No exclusion criteria applied. However those who could not read or write in French were not able to participate since the study questionnaire was only available in French.

### Outcome measures and data collection

The primary outcome, EMS calls (behaviors), was extracted from the EMS call center databases for the three periods. All calls concerning an adult aged 18 years or older, living in the studied county, and presenting FAST symptoms (Facial drooping, Arm or leg weakness, Speech difficulties) or sudden onset of neurological signs with no other immediate explanation, were included. Additionally, patients identified by the emergency dispatcher as stroke or suspected transient ischemic attack were also included. Patients’ socio-demographic data, clinical signs, hour of symptom onset, hour of EMS call, and management (triage and dispatch) were collected.

Secondary outcomes related to public attitudes and knowledge were assessed at T0 and T1 using a 3-section ad-hoc questionnaire on knowledge (stroke definition, risk factors, symptoms), attitudes (urgency of the situation, response to adopt), and socio-demographic data. The questionnaire was developed by the steering committee based in available literature [10–12]. Its acceptability and understandability were assessed on a sample of lay users. Questions included both multiple choice questions and open-ended questions. Questions concerning stroke knowledge were open-ended; participants were asked to define stroke and which symptoms were evocative of acute stroke. To assess attitudes and the appropriateness of the response, participants had to choose between five proposals (calling EMS, calling firefighters, going to the emergency unit, visiting a general practitioner, calling a relative) and between responding immediately or waiting to see if symptoms disappeared. The questionnaire was sent by postal and electronic forms in the Rhône and Isère counties to the beneficiaries of a private health insurance, members of a neurological disease foundation, and the union for small and medium businesses (*Confédération des petites et moyennes entreprises*). We also used informal spreading and snowballing through Facebook and the internet websites of the aforementioned partners. In line with the French regulation for health research that applied, no consent for participation in the study was required for the type of our study.

### Data analysis

Based on the data obtained from the EMS registries, the expected number of calls for stroke suspicions was 160 per 2-month period in the Isère county and 200 per 2-month period in the Rhône county. This ensured a statistical power of 95% to demonstrate a 10% absolute difference in the increase in the number of calls between the two counties over the study period. Quantitative variables were described by their mean and standard deviation or median and first and third quartiles (Q1-Q3), depending on their distribution. Qualitative variables were described using frequency and percentage.

The evolution of the main outcome (number of EMS calls) over the 3 periods was compared between the intervention and control counties using a Poisson regression analysis including as dependent variable number of EMS call, and as independent variables the county (Rhône / Isère) and the period (T0, T1 and T2). The time from symptom onset to EMS call was analyzed using a mixed linear regression model. The model included as dependent variable time from symptom onset to EMS call, and as independent variables age (< 65/≥65), gender (male/female), time of call (day 8 h–18 h/evening 18 h–22 h/night 22 h–8 h), and presence or absence of each symptom: facial paralysis, motor deficit, and speech disorder, with random effects on the intercept and the county to take into account the time correlations within counties.

Changes in public knowledge and attitudes between T0 and T1 were compared using multivariate mixed regression models with a random effect on the intercept and on the county. We estimated the impact of the campaign on the following outcomes, included as dependent variables in the models (one model per dependent variable): knowing at least two FAST symptoms (yes/no), knowledge of the emergency of the situation (yes/no), and knowledge of the need to call EMS (yes/no). Independent variables included in each model were gender (female/male), age (age under 45 years old/[45–64]/≥65), educational level (low (less than graduate)/medium (graduate to 4 years post graduate)/high (≥5 years post graduate)), and history of stroke (stroke survivor or relative of a stroke survivor/no stroke history). *P*-values were two-tailed and 0.05 was considered as significant, all confidence intervals were calculated at 95% (95%CI). Analyses were conducted using SAS software 9.3 for Windows (SAS Institute Inc., Cary, NC, USA).

## Results

### Impact on behaviors: EMS calls

During the 3 periods, 217,476 EMS calls were recorded in the Rhône county, of which 707 met the inclusion criteria and were included (n_T0_ = 214, n_T1_ = 234, n_T3_ = 259). In Isère, 74,526 EMS calls were recorded, 519 met the inclusion criteria and were included (n_T0_ = 186, n_T1_ = 174, n_T3_ = 159; Appendix 2).

Calls and patients’ characteristics are displayed in Table 1. Pooling the three time periods and the two counties (*N* = 1226 calls), mean+/−SD age of patients was 70.9+/− 17.9 years and 52.0% were women. More than 80% of included calls described a positive FAST score, main symptoms being speech disorder, and arm or leg weakness. The number of EMS calls for stroke suspicion increased by 21% between T0 (*N* = 214) and T2 (*N* = 259) in the Rhône county, while it decreased by 14% between T0 (*N* = 186) and T2 (*N* = 159) in Isère (*p* = 0.02 for Poisson regression analysis). There was no significant effect of the campaign on time from symptom onset to EMS call after adjustment on age, gender, symptoms described by the caller, and hour of call (evening+night vs. day; Table 2).

**Table 1:** Characteristics of EMS calls for stroke suspicion according to county and time period

	Rhône county (intervention)			Isère county (control)		
	T0 (*N* = 214)	T1 (*N* = 234)	T2 (*N* = 259)	T0 (*N* = 186)	T1 (*N* = 174)	T2 (*N* = 159)
**Age, yrs** (median [Q1-Q3])	75 [60–84]	73.5 [59–85]	75 [60–84]	77 [62–84]	77 [62–86]	76.5 [63.5–86]
**Gender**						
Female	105 (50.2)	131 (56.0)	150 (57.9)	76 (40.9)	90 (51.7)	83 (52.2)
Male	104 (49.8)	103 (44.0)	109 (42.1)	110 (59.1)	84 (48.3)	76 (47.8)
**Symptoms**						
Arm/leg weakness	119 (55.6)	106 (45.3)	105 (40.5)	125 (67.2)	91 (52.3)	89 (56.0)
Facial paralysis	54 (25.2)	62 (26.5)	77 (29.7)	71 (38.2)	65 (37.6)	56 (35.2)
Speech disorder	96 (44.9)	114 (48.7)	124 (47.9)	114 (61.3)	86 (49.7)	90 (56.6)
**Positive FAST score**	182 (85.0)	192 (82.0)	215 (83.0)	177 (95.0)	142 (81.6)	138 (86.8)
**Admission facility**						
SU	65 (31.5)	43 (19.0)	57 (22.7)	26 (14.0)	14 (8.0)	7 (4.4)
ED	132 (64.1)	168 (74.3)	182 (72.5)	157 (84.4)	151 (86.8)	148 (93.1)
ICU	4 (1.9)	2 (0.9)	0 (0.0)	0 (0.0)	3 (1.7)	1 (0.6)
Other	5 (2.4)	13 (5.7)	12 (4.8)	3 (1.6)	6 (3.4)	3 (1.9)

*EMS* Emergency Medical Services, *y* years, *SU* Stroke Unit, *ED* Emergency Department, *ICU* Intensive Care Unit

**Table 2:** Regression analysis for evolution of number of calls and time between symptom onset and call to EMS in the two counties

	Rhône county (intervention)			Isère county (control)			
	T0	T1	T2	T0	T1	T2	*p*-value*
Number of EMS calls for stroke suspicion	214	234	259	186	174	149	0.020
Time from symptom onset to EMS call (median [Q1-Q3])	30 [10–136]	60 [15–177]	42 [15–120]	42 [15–110]	36 [11–76]	36 [11–107.5]	0.928

*p-value for Poisson regression analysis for number of EMS call, and for multivariate mixed linear regression adjusted on age, gender, symptoms, and hour of call for time from symptom onset to EMS call

### Impact on public stroke knowledge and attitudes

Among the 1468 questionnaires received, 1421 could be included, 1183 in the intervention county and 238 in the control county (Appendix 3). Socio-demographic characteristics of respondents revealed differences between the 2 counties and the 2 time-periods regarding age, being a relative of a stroke survivor, and educational level (Table 3). In the Rhône and Isère counties, respectively, median age was 48 years at T0 and 36 years at T1, and 57 years at T0 and 49 years at T1. Proportion of women among respondents was in the Rhône and Isère counties, respectively, 71.26% at T0 and 74.22% at T1, and 70.97% at T0 and 75.86% at T1.

**Table 3:** Characteristics of questionnaire respondents according to county and time period

	Intervention county				Control county			
	T0 (*n* = 665)		T1 (*n* = 518)		T0 (*n* = 93)		T1 (*n* = 145)	
**Age, yrs (**median [Q1-Q3])	48	[34–62]	36	[24–48]	57	[36–68]	49	[28–70]
18–44 years old	298	(44.8)	364	(70.3)	31	(33.3)	70	(48.3)
45–64 years old	224	(33.7)	107	(20.7)	28	(30.1)	24	(16.6)
≥ 65 years old	143	(21.5)	47	(9.1)	34	(36.6)	51	(35.2)
**Gender**								
Male	190	(28.74)	133	(25.78)	27	(29.03)	35	(24.14)
Female	471	(71.26)	383	(74.22)	66	(70.97)	110	(75.86)
**Educational level**								
Low	117	(17.97)	74	(14.31)	23	(25.54)	53	(28.61)
Medium	390	(59.91)	328	(63.45)	42	(37.19)	49	(35.51)
High	144	(22.12)	115	(22.24)	24	(26.97)	36	(26.09)
**History of stroke**								
Stroke survivor	13	(2.00)	11	(2.12)	7	(7.61)	3	(2.08)
Relative of a stroke survivor	437	(66.82)	344	(66.54)	66	(74.16)	89	(63.57)
**Stroke risk factors**								
Low physical activity	72	(10.93)	51	(9.85)	20	(23.26)	24	(17.14)
Active smoker	95	(14.31)	104	(20.08)	14	(15.22)	20	(14.08)
Diabetes mellitus	32	(5.11)	16	(3.13)	7	(7.87)	21	(15.56)
Hypertension	98	(15.31)	56	(10.92)	13	(14.77)	34	(24.46)
Cardioembolic disease	12	(4.00)	4	(3.28)	3	(9.68)	7	(14.00)

In the intervention county (Rhône), knowledge of at least 2 stroke symptoms significantly increased from 40.5 to 48.7% between T0 and T1 (*p* = 0.038, Table 4). In this county, about 1 in 2 respondents knew a treatment existed but less than 10% mentioned thrombolysis or described such an acute phase treatment, and this was not improved after the campaign (*p* = 0.682). Knowledge that people of all ages could be stroke victims significantly improved after the campaign (20.1% at T0 to 38.6% at T1, *p* < 0.001).

**Table 4:** Stroke knowledge according to county and time period

	Intervention county					Control county				
	T0 (*n* = 665)		T1 (*n* = 518)		*p*-value	T0 (*n* = 93)		T1 (*n* = 145)		*p*-value
**Stroke warning signs**										
At least 2 FAST symptoms	269	(40.5)	252	(48.7)	**0.038**	43	(46.2)	71	(49.0)	0.606
All 3 FAST symptoms	52	(7.8)	55	(10.6)	0.167	5	(5.4)	13	(9.0)	0.220
Arm or leg weakness	337	(54.8)	282	(56.7)	0.897	49	(57.7)	74	(58.3)	0.944
Facial weakness	125	(20.3)	133	(26.8)	0.052	12	(14.1)	38	(29.9)	0.023
Speech disturbance	335	(54.5)	300	(60.4)	**0.010**	53	(62.4)	80	(63.0)	0.899
Balance disturbance	71	(11.5)	68	(13.7)	0.105	15	(17.7)	16	(12.6)	0.600
Visual disturbance	101	(16.4)	59	(11.9)	0.149	14	(16.5)	11	(8.7)	0.282
Coma	123	(20.0)	87	(17.5)	0.512	16	(18.8)	21	(16.5)	0.430
**Response when faced with stroke**										
Stroke is an emergency situation	660	(99.3)	511	(98.7)	0.241	90	(96.8)	142	(97.9)	0.257
EMS call	545	(82.0)	417	(80.5)	0.725	74	(79.6)	114	(78.6)	0.837
**Availability of a treatment for stroke**										
Yes	383	(58.6)	251	(48.6)	0.113	55	(61.1)	69	(48.6)	0.583
No	87	(13.3)	100	(19.3)		12	(13.3)	18	(12.7)	
Do not know	183	(28.0)	166	(32.1)		23	(25.6)	55	(38.7)	
**Knowledge of thrombolysis**^**a**^	21	(3.2)	14	(2.7)	0.682	2	(2.2)	9	(6.2)	0.084
**Age of stroke victims**										
Only older than 65	42	(6.40)	5	(1.0)	**0.002**	9	(9.9)	12	(8.5)	0.638
Only older than 18	457	(69.7)	263	(50.8)	**< 0.001**	54	(59.3)	85	(59.9)	0.337
All ages	132	(20.1)	200	(38.6)	**< 0.001**	26	(28.6)	36	(25.4)	0.605

^a^ Thrombolysis or description of an agent to dissolve thrombus

Results are presented as n(%). *P*-values after adjustment on age, gender, educational level, and history of stroke

In the control county (Isère), no significant changes happened between the 2 periods (Table 4). At T0 and T1, respectively 46 and 49% of respondents knew at least 2 FAST stroke symptoms. At T0 and T1, about 98% of respondents mentioned that stroke was an emergency, and 79% would call EMS (*p* > 0.05). Similarly to the intervention county, 48% declared that a treatment existed but very few described the existence of an acute phase treatment such as thrombolysis, with no significant difference between T0 and T1.

Multivariate analyses on knowledge of at least 2 stroke warning signs, knowledge of the emergency of the situation, and knowledge of the need to call EMS, revealed no significant difference in the knowledge and attitudes between the intervention and the control group, although there was a trend for a higher improvement in the intervention county regarding knowledge of at least 2 stroke warning signs (OR [95%CI] = 1.23 [0.90–1.67], *p* = 0.160). After adjustment, factors associated with increased knowledge were being a woman (OR [95%CI] = 1.49 [1.15–1.93], *p* = 0.002), having a high educational level compared to a low educational level (OR [95%CI] = 3.03 [2.13–4.31], *p* = 0.001), and being a stroke survivor or a relative of a stroke survivor (OR [95%CI] = 1.57 [1.24–2.58], *p* = 0.002) (Appendix 4).

## Discussion

After implementation of a local campaign on stroke, the number of EMS calls for stroke suspicion significantly increased at 12 months in the intervention county compared to the control county. Nevertheless, no significant improvement in time from symptom onset to EMS call was observed, and the increase in symptom knowledge was not significantly different between intervention and control.

The present theory-informed campaign was associated to a significant improvement in behaviors, indicating that the messages conveyed might have improved individuals’ skills to call EMS when faced with stroke. In a review on stroke campaign effects, Mellon et al. [11] showed that the strongest results on behaviors were obtained after repeated and sustained campaigns [17, 18] or campaigns also targeting health professionals [19]. The lack of repetition over time may explain why the present campaign failed to increase other outcomes such as time from symptom onset to EMS call. The first message of the campaign was to call EMS, hence repetition might have facilitated integration of the other messages.

In the existing literature, other national or local experiments have shown improvements in the population’s knowledge [20–23] and/or behaviors (i.e. shorter patient delay, higher EMS or ambulance use, higher thrombolysis rate) following information campaigns [11, 18]. In the present study, knowledge of stroke warning signs significantly increased in the Rhône county after the campaign, however it remained low. The absolute 8% increase observed herein is similar to that observed in Canada and Australia, but the percentage of people knowing at least 2 stroke warning signs was substantially higher in the latter studies than in the current one [20]. Attitudes in case of acute stroke, i.e. calling EMS immediately, did not increase in the present sample but was already very high at baseline before the campaign. However, the gap between knowledge, intention, and behavior still needs to be bridged. Another interesting finding is that knowledge concerning thrombolysis appears to be highly confidential among respondents, which is in line with what has been observed in other countries [23]. The spreading and efficacy of thrombolysis, and more recently endovascular thrombectomy, represent an opportunity to better communicate on the therapeutic options for stroke management. Messages of future stroke campaign should focus on treatment and emphasize the benefits of receiving them rapidly.

We observed that women, people with a higher level of education, and stroke survivors or their relatives had significantly better knowledge of stroke. However, no difference was observed for respondents with risk factors. These results point out the necessity to complete mass campaigns with actions targeting specific subgroups, such as people at risk or people with low socio-educational level, to reduce inequalities. Stroke information programs with actions targeting specific groups have shown positive effects in disadvantaged counties [24, 25]. Campaigns must offer various supports specifically tailored to different levels of health literacy and use a variety of media to reach all audiences.

There are some limitations to this work. Missing data on the time of symptom onset limited the possibility to analyze the effect of the campaign on this parameter. This limitation is due to the data collection process of the EMS call registry which is integrated into usual care. This reflects a need for improving EMS dispatchers’ practices, as symptom onset hour is a key factor for acute stroke management. Another limitation concerns a potential selection bias inherent to the sampling method that led to differences in the demographic characteristics of the respondents from the two counties compared with census data and between the 2 periods within each county. We took this limit into account by adjusting the analyses on potential confounding factors. The study is also limited to assessing the influence of the intervention on the intention to call EMS. However, the study did not evaluate the relationship between the outcome variables and the TPB, and the influence of the intervention on the individual constructs of the TPB as applied to this study population.. Individual repeated measures would be interesting to in-depth changes induced by the campaign at individual level. This study presents also several strengths. Indeed, few information campaigns were evaluated with a sufficient level of evidence and a control group [10, 23]. Mellon et al. pointed out a large heterogeneity among interventions, evaluation designs, and effect sizes of the interventions between the campaigns that have been evaluated [11]. Another strength is the user-centered and collaborative approach, involving representatives of all stake-holders and the use of a theoretical model [14]. The latter helped us encompass barriers such as the lack of perceived control and of perceived benefit to call EMS, previously identified [13]. This campaigned could easily be generalized and reproduced in different settings. Moreover, our evaluation measured outcomes related to both behaviors, attitudes, and knowledge with a 12-month perspective. While this study provided a quantitative evaluation of the campaign, associating qualitative approach might be useful to understand how the campaign impact behaviors in the light of the TBP.

In France, a national campaign on stroke is launched each year [26], but there is no structured display with massive broadcasting covering the entire territory, leading to the development of local initiatives [17]. The literature advocates for campaigns that are broadcasted on television, with important financial support [20]. It would be relevant to propose in France a more structured, large-scale national program, displayed on mass media with large audiences, and to ensure local proximity back-up with actions targeting specific populations such as high-risk patients or underprivileged populations. This is even more crucial since a recent study showed territorial disparities in stroke incidence and mortality [27, 28].

## Conclusions

The local stroke information campaign put in place herein increased EMS call behavior and, to a lesser extent, knowledge concerning stroke warning signs. Increase in EMS for stroke suspicion constitutes the first step of the acute phase of stroke care, this might thus lead to increase in access to reperfusion strategies. Our results advocate for the design of more sustained and actively broadcasted campaigns to rise stroke awareness on effective treatments. The revolution in acute stroke management due to the development of mechanical thrombectomy constitutes an opportunity to rethink new and more powerful messages.

## Supplementary Information

**Additional file 1: Appendix 1.** Campaign resources. **Appendix 2.** Flow chart for inclusion of EMS calls. **Appendix 3.** Flow chart for inclusion of questionnaires on stroke knowledge and attitudes. **Appendix 4.** Multivariate logistic regression analysis for changes in stroke knowledge and attitudes between T0 and T1 (*N* = 1421).

## Data Availability

The datasets analysed during the current study available from the corresponding author (at Hospices Civils de Lyon) on reasonable request.

## Abbreviations

ED: Emergency Departments
EMS: Emergency Medical Services
ICU: Intensive Care Unit
SU: Stroke Unit
