# Supplementary material for: Impact of a theory-informed and user-centered stroke information campaign on the public’s behaviors, attitudes, and knowledge when facing acute stroke: a controlled before-and-after study
Source: BMC Public Health. 2020 Nov 16;20:1712. doi: 10.1186/s12889-020-09795-y (PMC7667807; doi:10.1186/s12889-020-09795-y)
Supplement: Supplementary file 1 — Additional file 1: Appendix 1. Campaign resources. Appendix 2. Flow chart for inclusion of EMS calls. Appendix 3. Flow chart for inclusion of questionnaires on stroke knowledge and attitudes. Appendix 4. Multivariate logistic regression analysis for changes in stroke knowledge and attitudes between T0 and T1 (N = 1421). [file 12889_2020_9795_MOESM1_ESM.docx]

Supplemental online appendix

Impact of a theory-informed and user-centered stroke information campaign on the public’s behaviors, attitudes, and knowledge when facing acute stroke - Haesebaert J et al

Appendix 1: Campaign resources

1a. Campaign posters (3 visuals)

*All individuals on campaigns materials provided in appendix of this publication have given their written consent for their likeness to be displayed in these images.*

*All individuals on campaigns materials provided in appendix of this publication have given their written consent for their likeness to be displayed in these images.*

*All individuals on campaigns materials provided in appendix of this publication have given their written consent for their likeness to be displayed in these images.*

1b. Information leaflet

*All individuals on campaigns materials provided in appendix of this publication have given their written consent for their likeness to be displayed in these images.*

1c. Website

- url <http://avc.univ-lyon1.fr/>

- screen shot


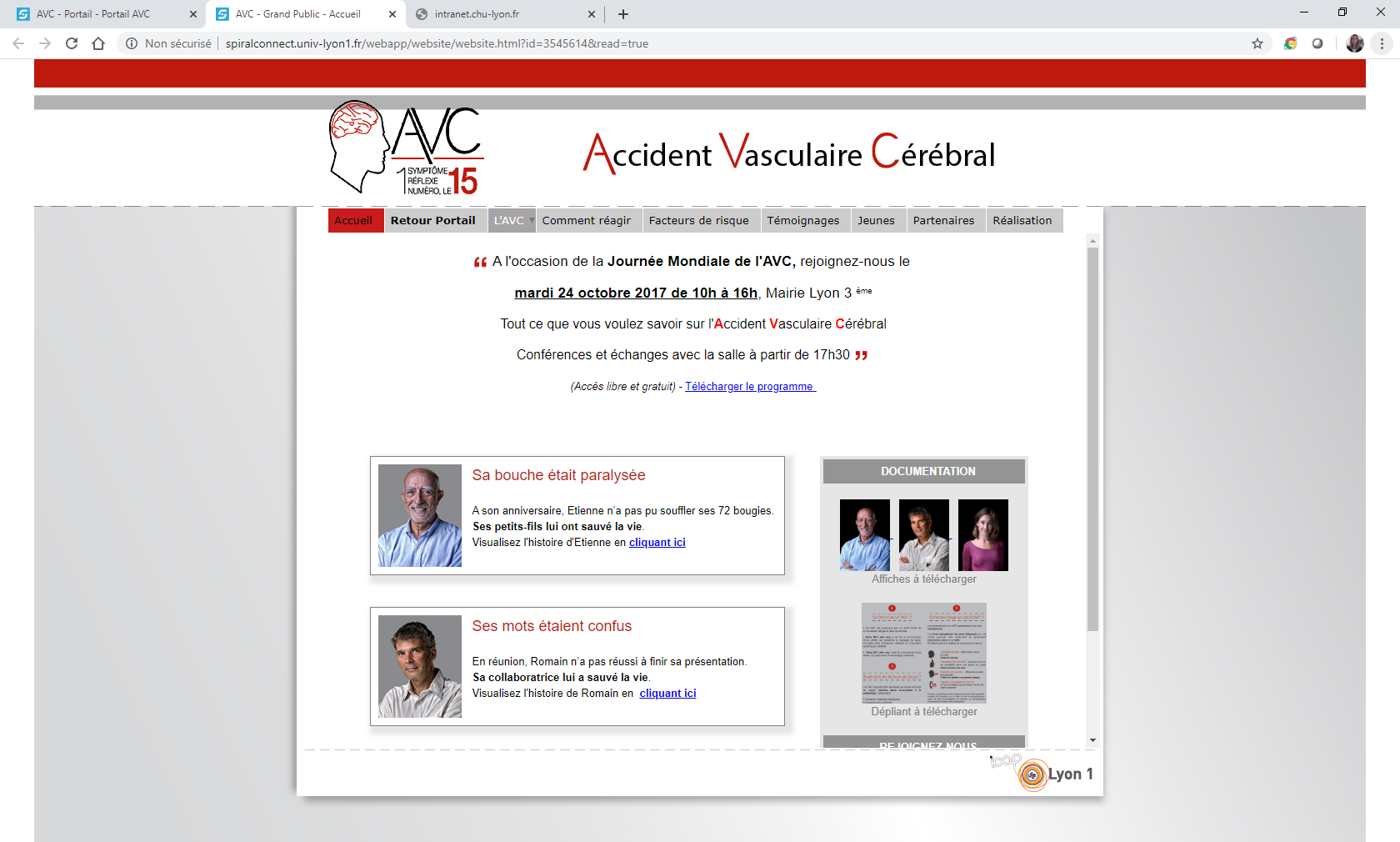


*All individuals on campaigns materials provided in appendix of this publication have given their written consent for their likeness to be displayed in these images.*

| 217,476 EMS calls  1,404 EMS calls with stroke symptoms  707 included EMS calls  T0: 214  T1: 234  T2: 259  197 duplicates  37 aged under 18 yo  463 other direct etiology  No stroke symptoms 216,072 | 74,526 EMS calls  527 EMS calls with  stroke symptoms   1. included EMS calls   T0: 186  T1: 174  T2: 159  6 duplicates  2 aged under 18 yo  No stroke symptoms 73,999 |
| --- | --- |
| 1a: Flow-chart for the Rhône county | **1b Flow chart for the Isère county** |

Appendix 2: Flow chart for inclusion of EMS calls

Appendix 3: Flow chart for inclusion of questionnaires on stroke knowledge and attitudes

Appendix 4: Multivariate logistic regression analysis for changes in stroke knowledge and attitudes between T0 and T1 (N=1421)

|  | ≥2 FAST warning signs | | EMS call in case of stroke | | Emergency situation | |
| --- | --- | --- | --- | --- | --- | --- |
|  | OR [95%CI] | p-value | OR [95%CI] | p-value | OR [95%CI] | p-value |
| Gender |  |  |  |  |  |  |
| Male | 1 |  | 1 |  | 1 |  |
| Female | 1.49 [1.15-1.93] | **0.002** | 0.85 [0.62-1.17] | 0.327 | 1.49 [0.56-4.01] | 0.425 |
| Age class |  |  |  |  |  |  |
| 18-44 years old | 1 | 0.310 | 1 | 0.293 | 1 | 0.642 |
| 45-64 years old | 1.14 [0.87-1.50] |  | 1.25 [0.89-1.77] |  | 0.99 [0.26-3.88] |  |
| ≥65 years old | 0.87 [0.61-1.24] |  | 1.35 [0.87-2.10] |  | 0.57 [0.15-5.53] |  |
| Educational level |  |  |  |  |  |  |
| Low | 1 | **<0.001** | 1 | **0.029** | 1 | 0.318 |
| Middle | 1.79 [1.24-2.58] |  | 1.42 [0.94-2.16] |  | 2.80 [0.52-7.40] |  |
| High | 3.03 [2.13-4.31] |  | 1.73 [1.50-2.60] |  | 2.12 [0.73-10.75] |  |
| Stroke survivor or relative of a stroke survivor | 1.57 [1.24-2.58] | **0.002** | 0.86 [0.64-1.16] | 0.317 | 2.12 [0.81-5.53] | 0.124 |
| Campaign (time*area) | 1.23 [0.90-1.67] | 0.160 | 0.94 [0.65-1.36] | 0.821 | 0.98 [0.55-2.73] | 0.157 |

Analysis was conducted using the GLIMMIX procedure (SAS software). We modeled the impact of the campaign (interaction between area and time) on knowing at least 2 symptoms of the FAST message (yes/no), knowledge concerning the emergency of the situation (yes/no), and knowledge concerning the need to call EMS (yes/no)
